# Climate change and its influence on the Descompensation of Affective Disorders

**DOI:** 10.1192/j.eurpsy.2026.11104

**Published:** 2026-07-31

**Authors:** E. Manrique Astiz, S. Yurrita Montesinos, A. Ballesteros Prados, A. López Capapé

**Affiliations:** 1 Psychiatry, Navarra Mental Health Rehabilitation Unit, Pamplona; 2 Psychiatry, Donostia University Hospital, Donostia; 3 Psychiatry, Tudela Mental Health Center, Tudela, Spain

## Abstract

**Introduction:**

Climate change represents a global health challenge with both direct and indirect effects on mental health. Seasonal affective patterns in mood disorders, especially bipolar and major depressive disorders, are influenced by temperature, luminosity, and seasonal transitions. Recent climatic variability may be altering the traditional timing of affective decompensations, increasing clinical unpredictability.

**Objectives:**

To explore whether patients with affective disorders perceive changes in their affective alterations compared with those they experienced or perceived over the past five years, and whether they associate these changes with climatic variations.

**Methods:**

A qualitative study using Grounded Theory was conducted in Zone IV of Tafalla Mental Health Services (Navarra, Spain). Semi-structured interviews were carried out with 10 euthymic patients with a history of affective disorders and seasonal patterns. Qualitative studies typically rely on smaller, theory-driven samples rather than large quantitative cohorts; theoretical sampling was applied until saturation. Transcripts were analyzed using open, axial, and selective coding with ATLAS.ti® 7. Triangulation strategies and memoing ensured analytic rigor, and the study underwent validation, which was deemed adequate.

**Results:**

Participants described perceived changes in the onset and duration of affective episodes, wihich increasingly occurred earlier than in previous years. They linked these shifts to hotter summers, milder winters, irregular rainfall, and altered light patterns. A central theme emerged: climate variability modifies the environmental cues that previously structured their illness rhythms. Patients reported adaptive strategies but also feelings of uncertainty and loss of control.

**Conclusions:**

Patients perceive climate change as influencing the timing of their affective episodes. These findings suggest that environmental variability may disrupt established seasonal patterns, requiring adaptive preventive and therapeutic strategies in mental health care. Qualitative methods offer valuable insight into how global climatic changes manifest in individual illness trajectories.

Keywords: Climate change;seasonal affective disorder;bipolar disorder;qualitative research;Grounded Theory;

**Disclosure of Interest:**

None Declared

